# Current Status of Stem Cell-Derived Therapies for Parkinson’s Disease: From Cell Assessment and Imaging Modalities to Clinical Trials

**DOI:** 10.3389/fnins.2020.558532

**Published:** 2020-10-16

**Authors:** Se Eun Jang, Lifeng Qiu, Ling Ling Chan, Eng-King Tan, Li Zeng

**Affiliations:** ^1^Neural Stem Cell Research Lab, Research Department, National Neuroscience Institute, Singapore, Singapore; ^2^Department of Diagnostic Radiology, Singapore General Hospital, Singapore, Singapore; ^3^Neuroscience & Behavioral Disorders Program, Duke University and National University of Singapore (DUKE-NUS), Graduate Medical School, Singapore, Singapore; ^4^Department of Neurology, National Neuroscience Institute, Singapore General Hospital Campus, Singapore, Singapore; ^5^Lee Kong Chian School of Medicine, Nanyang Technological University, Novena Campus, Singapore, Singapore

**Keywords:** Parkinson’s disease, dopaminergic neurons, transplantation, stem cells, imaging modalities, neuroimaging, clinical trials

## Abstract

Curative therapies or treatments reversing the progression of Parkinson’s disease (PD) have attracted considerable interest in the last few decades. PD is characterized by the gradual loss of dopaminergic (DA) neurons and decreased striatal dopamine levels. Current challenges include optimizing neuroprotective strategies, developing personalized drug therapy, and minimizing side effects from the long-term prescription of pharmacological drugs used to relieve short-term motor symptoms. Transplantation of DA cells into PD patients’ brains to replace degenerated DA has the potential to change the treatment paradigm. Herein, we provide updates on current progress in stem cell-derived DA neuron transplantation as a therapeutic alternative for PD. We briefly highlight cell sources for transplantation and focus on cell assessment methods such as identification of genetic markers, single-cell sequencing, and imaging modalities used to access cell survival and function. More importantly, we summarize clinical reports of patients who have undergone cell-derived transplantation in PD to better perceive lessons that can be drawn from past and present clinical outcomes. Modifying factors include (1) source of the stem cells, (2) quality of the stem cells, (3) age of the patient, (4) stage of disease progression at the time of cell therapy, (5) surgical technique/practices, and (6) the use of immunosuppression. We await the outcomes of joint efforts in clinical trials around the world such as NYSTEM and CiRA to further guide us in the selection of the most suitable parameters for cell-based neurotransplantation in PD.

## Introduction

Parkinson’s disease (PD) is one of the most prevalent chronic neurodegenerative disorder characterized by the selective, progressive loss of nigrostriatal dopaminergic (DA) neurons in the substantia nigra pars compacta. The main hallmarks of PD include the presence of α-synuclein positive Lewy bodies and neuroinflammation (MacGeer and McGeer, 2008; More et al., 2013) that extends across many areas of the central nervous system (CNS), affecting the enteric and autonomic systems, in particular (Goedert et al., 2013), causing impairments in motor movements such as bradykinesia (slowed movements), tremors, postural instability, and muscle rigidity. Furthermore, PD patients have shown non-motor disease manifestations such as rapid eye movement (REM), sleep behavior disorders, depression, hyposmia, and constipation (Olanow et al., 2009). Unfortunately, there are no curative therapies available to modify or reverse the progression of the underlying disease processes to date.

The current gold standard for PD treatment is through the ingestion of levodopa, which has been approved by the US Food and Drug Administration in the 1970s and has continuously shown positive results in temporal amelioration of PD symptoms (Fahn, 2003, 2006). However, long-term exposure to levodopa results in a gradual decrease in drug effectiveness and shorter periods of benefit, leading to levodopa-induced dyskinesias (motor fluctuations), as well as psychiatric and cognitive problems. Alternatively, surgical strategies, such as deep brain stimulation (DBS), have shown to alleviate PD motor symptoms (Siegfried and Lippitz, 1994; Limousin et al., 1995) and offer symptomatic relief that cannot be controlled with medications (Alamri et al., 2015). However, its application is not only limited to early-to-mid PD stages but also loses efficacy after a few years (deSouza et al., 2013).

In the last few decades, cell-based therapy using human stem cells has made large strides in overcoming the abovementioned limitations in PD treatment. Also known as regenerative medicine, stem cell therapy is believed to replace diseased, dysfunctional, or damaged tissue in hopes to restore lost neuronal circulatory caused by focal degeneration of mesencephalic dopaminergic (mDA) neurons. Specifically, neural progenies from pluripotent stem cells (PSCs) are known to hold great potential as a succeeding treatment for neurodegenerative diseases (Hu et al., 2010; Kriks et al., 2011; Ma et al., 2012). Today, DA neurons differentiated from stem cells are paving the way as a new, alternative approach in the treatment of PD. In this review article, we briefly highlight the major sources of stem cells used in preclinical and clinical PD observations (have been thoroughly reviewed in various articles, refer to Stoker, 2018). We focus on key methodologies currently applied in cell assessment, imaging modalities, and also further discuss ongoing stem cell-based clinical trials in PD. This also includes key challenges that the field is encountering and the prospects of stem cell therapy in PD.

## Cell Sources

First, we briefly discuss the various types of stem cells currently being used as a source for cell-based therapy in PD. We also include the pros and cons of each cell line (Table 1), followed by the characterization of graft quality through various cell assessment methods (Cell Assessment of Differentiated DA Neurons section).

**TABLE 1:** Cells used in transplantation for Parkinson’s disease (PD).

**Cell type**	**Clinical trial**	**Advantages**	**Disadvantages**
Fetal ventral mesencephalic cells (fVM)	Yes/ongoing (TRANSEURO; NCT01898390)	• Good long-term graft survival post-transplantation	• Unpredictable and limited supply of cell source Ethical concerns
Embryonic stem cells (ESC)	Ongoing (European-based STEM-PD, NYSTEM, NCT02452723, NCT03119636)	• Indefinite expandability • Good graft survival post-transplantation • Advancement in GMP-grade cells	• Ethical concerns • Possible risk of tumorigenesis • Tissue rejection; pre- and post-operative immunosuppression
Induced pluripotent stem cells (iPSC)	Yes/ongoing (CiRA)	• Indefinite expandability • Easily accessible cell source • Immunologically matching cells • No need of immunosuppression treatment	• High heterogeneity of cell line between individual cell line resulting in complex procedures • Low reprogramming efficiency • High operative cost • Time consuming • Possible risk of tumorigenesis
Neural progenitor cells (NPC)	Yes/ongoing (NCT03309514, NCT01329926)	• Multipotent cells • Easy expansion and differentiation protocol • Large quantity	• Invasive tissue collection step • Limited proliferation • Low graft survivability • Limited proliferative ability

### Fetal Ventral Mesencephalic Cells

In the early 1970s, Olson and colleagues successfully transplanted adrenal chromaffin cells and embryonic DA neurons into the anterior chamber of the eye in rats and showed that the viability of grafted neurons was best achieved using developing embryonic neurons (Olson and Malmfors, 1970; Olson and Seiger, 1972). Parkinsonism rat and monkey models grafted with early gestational age dopamine-rich mesencephalic neurons formed neurite protrusions and synthesize dopamine (Dunnett et al., 1983; Brundin et al., 1986; Redmond et al., 1986; Stromberg et al., 1986; Bakay et al., 1987). Furthermore, successful integration of transplanted cells into the host brain neuronal network was demonstrated through synaptic integration using a rabies-based monosynaptic tracing method (Cardoso et al., 2018). Behavioral studies in PD rodents and primates with human fetal DA neuron transplantation showed higher efficacy in improvement of behavioral deficits as compared to conventional adrenal medullary tissue transplants (Bjorklund and Stenevi, 1979, Perlow et al., 1979; Freed et al., 1981; Morihisa et al., 1984). Also, pioneering clinical studies in human fetal ventral mesencephalic (fVM) transplantation into the caudate and putamen of PD patients in Sweden, United Kingdom, and United States reported moderate amelioration of PD symptoms (Lindvall et al., 1988; Madrazo et al., 1988; Freed et al., 1990; Freed et al., 2001; Olanow et al., 2003). Moreover, normal striatal F-DOPA uptake was 3–5 years post-surgery, including gradual motor improvements that sustained up to 18 years post-transplantation (Kefalopoulou et al., 2014). However, the majority of successful cases were performed in PD patients under the age of 60 (Ma et al., 2010). Whether graft-induced dyskinesias are characteristics of neural transplantation has to be better studied and analyzed (Freed et al., 2001; Hagell et al., 2002; Olanow et al., 2003; Ma et al., 2010). Nonetheless, obtaining as many as up to seven human fetal donors (aged 6–9 weeks after conception) for each host raises many ethical concerns and logistical challenges for a disease affecting millions of people worldwide (Steinbeck and Studer, 2015; Barker and Consortium, 2019). Furthermore, the difficulty in preaccessing a cell type before transplantation is a major challenge in standardization as the heterogenicity of cell population within the graft is inevitable, contributing to high variability in the degree of symptomatic recovery. All in all, the additional risk in cell contamination of unwanted cell types during tissue extraction hampered the downstream translation of fVM transplantation as an alternative therapeutic option.

### Human Embryonic Stem Cells

Due to the abovementioned ethical controversies in utilizing hfVM tissues for cell-based therapy (and other limitations), human embryonic stem cells (hESCs) were identified as a prospective substitute (Thomson et al., 1998; Reubinoff et al., 2000; Barker, 2014). These subsets of pluripotent cells are located in the inner cell mass of early embryonic blastocyst commonly derived from *in vitro* fertilization (Evans and Kaufman, 1981; Thomson et al., 1998) and hold the capability to generate into a plethora of cell lines through a spontaneous differentiation protocol *in vitro* (Itskovitz-Eldor et al., 2000; Lee et al., 2000; Reubinoff et al., 2001; Zhang et al., 2001). In the case of neuroepithelial cell-derived DA neuron differentiation, cells showed an increase in a multitude of cellular marker expression for midbrain DA neurons with fiber outgrowth (Thomson et al., 1998; Kawasaki et al., 2000; Kim et al., 2002) and electrophysiologically active neurons that produced DA in an activity-dependent manner (Yan et al., 2005). In later years, it was identified that DA neurons unlike all other neurons are generated from the midbrain floor plate. With newly improvised DA neuron differentiation protocol (Fasano et al., 2010; Kriks et al., 2011; Kirkeby et al., 2012), a significant upregulation of midbrain DA neuronal markers was observed along with recovery in motor defects in preclinical studies (Kirkeby et al., 2012, 2017a; Grealish et al., 2014). Unfortunately, key limitations lie in the difficulty in controlling the maturation stage of embryonic cultures and cellular heterogeneity, which may lead to negative outcomes in therapeutic applications (Stewart et al., 2006; Roy et al., 2006; Cho et al., 2008; Koch et al., 2009). Other caveats include the associated risk in tumor generation and teratoma due to their high pluripotent phenotype (Ben-Hur et al., 2004; Roy et al., 2006; Brederlau et al., 2006; Sonntag et al., 2007; Yang et al., 2008). In 2001, ethical concerns in hESC research resulted in a restriction on federal fundings in the United States. Fortunately, this legislation has been revoked by President Barack Obama in 2007. With this advantage, New York Stem Cell Science Consortia at Memorial Sloan Kettering Cancer Center conducted ongoing projects such as the development of good manufacturing practice (GMP) clinical-grade hESC-derived DA neurons for FDA approval in future transplantation studies (refer to section “GMP cryopreservation of cells”), optimization of cell purification to enrich A9 type DA neurons, and also, active involvement in strategical planning for clinical trial of hESCs in Parkinson’s disease.^1^

### Human-Induced Pluripotent Stem Cells (hiPSCs)

The field of stem cell research and regenerative medicine was revolutionized in 2006 when human fibroblast cells were successfully reprogrammed into pluripotent cell lines using four transcription factors: c-Myc (or Nanog, Lin28), Oct3/4, Klf4, and Sox2 (Takahashi and Yamanaka, 2006; Takahashi et al., 2007; Yu et al., 2007). Reprogrammed iPSCs have been a highly attractive cell source as they have the characteristics of hESCs (in terms of morphology and genetic profile) (Fairchild, 2010; Phanstiel et al., 2011), and they have a relatively simpler extraction process. Tissue collection is non-invasive as host cells from skin fibroblast (Pulecio et al., 2014), peripheral blood mononuclear cells, and umbilical cord mesenchymal cells (Park et al., 2008; Senju et al., 2011; Biju et al., 2013; Qin et al., 2013) could be used to differentiate into patient-specific neurons *in vitro* (Soldner et al., 2009; Beevers et al., 2013; Eigentler et al., 2013; Sison et al., 2018). This would also avoid allogenic recognition and ethical concerns (Takahashi and Yamanaka, 2016). In PD studies, the quality of iPSC-derived DA neurons was highly similar to that of hESCs (Cooper et al., 2010; Doi et al., 2014; Kikuchi et al., 2017; Lehnen et al., 2017), and human leukocyte antigen (HLA)-matched allogeneic neural transplantation into monkeys increased the efficacy of cell survival and function (Morizane et al., 2017). Animal studies demonstrated successful amelioration of PD symptoms resulting from iPSC-derived DA neuron transplantation (Wakeman et al., 2017). Further refinement and characterization are necessary to achieve precise cell fate conversion of reprogrammed cells. Similar to ESCs, it is important that minimal manipulation is made during reprogramming prior to cell delivery.

### GMP Cryopreservation of Cells

The generation of good manufacturing practice (GMP)-compliant, deliverable midbrain DA (mDA) progenitors/neurons optimized for cell-based therapy for PD is a major challenge. Currently, a diverse collection of clinical-grade hESC lines are available as starting material to generate GMP-compliant mDA progenitors/neurons. In fact, GMP compliant differentiation protocols and reagents have been successfully applied to generate GMP mDA neurons (Liu et al., 2013; Peng et al., 2014).

In comparison, the availability of clinical-grade iPSCs is relatively lesser due to the lack of technology that involves complex reprogramming methodologies. Major hurdles of the clinical translation of mDA cells therapy include (i) quality control of the identity, safety, and efficacy of cell product in a consistent and real-time manner, (ii) determination of the precise time points at which DA precursors/neurons can be cryopreserved and banked without affecting its’ quality, (iii) good postthaw viability of mDA cells, and (iv) characteristics and functionality of the population of cells should have minimal to no alterations after thawing. XCell Science has generated GMP-compatible authentic DA neurons, which are functional when transplanted into PD animal model (Peng et al., 2014) where cells were cryopreserved at day 14 after neuronal stem cell (NSC) stage. Similar studies were also reported by Cellular Dynamics International using more mature mDA cells in postmitotic stage (Wakeman et al., 2017). Successful generation of GMP-grade cryopreserved cells would allow for storage of a large batch of DA neurons and also increase the flexibility in operational schedule organization without the dependence on GMP-manufacturing site.

## Cell Assessment of Differentiated DA Neurons

Understanding the key type of DA neurons required to achieve downstream restoration of PD pathology is essential. The mesotelencephalic DA system in the midbrain contains two main groups: the A9 neuronal clusters of the nigrostriatal DA pathway located in the zona compacta, the substantia nigra involved in the control of posture, and the A10 neurons located in the ventromedial mesencephalic tegmentum that regulates the locomotor activity and emotional behavior (Dahlstroem and Fuxe, 1964; Anden et al., 1966; Ungerstedt, 1971; Lindvall and Bjorklund, 1974; Pijnenburg et al., 1976; Papp and Bal, 1986). Dysfunction of the nigrostriatal system has been linked to Parkinsonism and later to schizophrenia, drug addiction, and depression (Robinson and Berridge, 1993; Meyer-Lindenberg et al., 2002). Differences between the two DA cell populations have been observed in neurochemistry and in spontaneous neuronal firing (Grenhoff et al., 1988; Wolfart et al., 2001; Neuhoff et al., 2002). More importantly, A9 neurons display significantly enhanced levels of neuromelanin pigmentation as compared to other dopamine-producing neurons (Mann and Yates, 1983; Hirsch et al., 1988; Gibb, 1992; Kastner et al., 1992). This could account for the association of early loss of A9 DA neurons in Parkinson’s disease with increased vulnerability upon disease progression with the relative preservation of A10 DA neurons (Hirsch et al., 1988; German et al., 1989; German et al., 1992; Damier et al., 1999; Halliday et al., 2005; Alavian et al., 2008).

Generally, stem cells are differentiated into specific nigra A9 DA neurons in large quantities prior to PD transplantation. This step has been thoroughly reviewed by many articles such as in Fan et al. (2020) and, thus, will not be further discussed here. However, we focus on developments in technology in cell assessment of differentiated DA neurons.

### Assessment of the Efficacy of Cell Transplants With Immunostaining Characterization

Prior to stem cell transplantation, it is important to be able to fully characterize differentiated cell types to avoid heterogenicity of cell population (also known as cellular contamination). Previous studies have shown that transplantation of fetal SN-A9 DA neurons suffices the requirement for striatal reinnervation and recovery of PD-like behavioral observations (Grealish et al., 2010). However, tumor formation (Roy et al., 2006; Brederlau et al., 2006; Elkabetz et al., 2008; Doi et al., 2012) and development of graft-induced dyskinesia could arise from the high heterogenicity of serotonergic neurons (Carlsson et al., 2007; Politis et al., 2010). As cells are normally transplanted as immature progenitor cells, developing methods that can characterize and predict its functional maturation and therapeutic efficacy is crucial. Hence, to circumvent these limitations prior to proceeding into clinical trials, methods to isolate homogenous population of DA progenitor cells have been closely evaluated (Fukuda et al., 2006; Pruszak et al., 2009; Jonsson et al., 2009; Ganat et al., 2012; Sundberg et al., 2013). This includes developing meaningful quality control assays to assess cell type to avoid having heterogeneous mixtures of cells (includes phenotypes and degree of maturity) and batch-to-batch variation. The quality of differentiated mesencephalic A9 DA neurons that represent those in the substantia nigral para compacta or into immature progenitor cells is vital to determine the therapeutic efficacy of cell transplantation in the Parkinsonian brain. It is well understood that the orchestration of specific gene expression patterns is highly correlated to DA cell differentiation and survival. Therefore, the establishment and determination of specific gene expression markers have been used to positively characterize differentiated cells *in vitro*.

In the case of mDA progenitor neuron specifications, positive gene expression of common transcription factors FOXA2, LMX1A, and OTX2 and negative markers (non-neural) such as Afp, Gata4, and Brachyury have been quantitatively analyzed (Chung et al., 2009; Lin et al., 2009; Jaeger et al., 2011; Kriks et al., 2011; Kirkeby et al., 2012; Salti et al., 2013; Doi et al., 2014). More importantly, the upregulation and downregulation of these markers at a given stage *in vitro* governs the efficiency of cell fate determination. Unfortunately, these markers have been shown to coexpress in the diencephalic progenitor cells of the subthalamic nucleus (STN) (Kee et al., 2017). Furthermore, the expression of the positive genetic marker for DA neurons, tyrosine hydroxylase (TH), a rate-limiting enzyme in dopamine synthesis (Daadi and Weiss, 1999; Sonntag et al., 2004; Kirkeby et al., 2017a), and the levels of GIRK2 have also been observed in many cell types *in vitro* (Thompson et al., 2005; Kirkeby et al., 2012; Reyes et al., 2012; Grow et al., 2016). Moreover, common positive markers used to isolate high-quality DA progenitor cells include EN1 and SPRY1 (Simon et al., 2001; Alberi et al., 2004; Kirkeby et al., 2017a); Nurr1 (Le et al., 1999); FOXA2, LMX1B, and MSX1 (Andersson et al., 2006; Chung et al., 2011), and the bicoid-related homeodomain factor Ptx3/Pitx3 (Hargus et al., 2010). It is noteworthy that some discrepancies have been found with the requirement for the presence of floor plate-specific cell surface marker CORIN expression (Ono et al., 2007; Chung et al., 2011; Kriks et al., 2011; Kirkeby et al., 2012, 2017a; Doi et al., 2014; Arenas et al., 2015; Fan et al., 2020). A more recent study has identified a cell surface marker integrin-associated protein (IAP, CD47) as a positive marker for FOXA2-positive DA progenitor cells (Lehnen et al., 2017).

While these positive markers are required to narrow down the search for pure DA progenitor cells, negative markers such as Oct3/4, PAX6, and SOX1 for other midbrain neurons act as good controls to prevent introducing contamination with other neuronal subtypes during sorting. Last, terminal differentiation of DA neurons post-transplantation can be identified by the expression of neurotransmitter phenotype markers, namely, TH, dopamine transporter (DAT), Vmat2, Girk2, and Calbindin (Di Porzio et al., 1990; Sgado et al., 2006). It is crucial to take into consideration the wide genetic variation of iPSCs, which may harbor a large spectrum of genetic variation and even retain donor-specific gene expression pattern depending on multiple factors, such as the number of passages of the lineage or transcriptional factors introduced to induce cell differentiation (Rouhani et al., 2014; Thomas et al., 2015; Burrows et al., 2016; Carcamo-Orive et al., 2017). Nonetheless, growing evidence strongly suggests the need for heightened stringency in cell type evaluation. This is particularly important to avoid incomplete differentiation of cells, which could result in undesired reprogrammed cell lineages affecting functional deficits when transplanted into PD models (Park et al., 2005; Grow et al., 2016; Kirkeby et al., 2017a).

### Single-Cell RNA-Seq to Evaluate the Quality of Cells

More recently, high-resolution analyses of cell type specificity such as single-cell transcriptomic analyses of neuronal populations of induced stem cells have pathed its way to become a new tool to increase the specificity during DA neuron extraction. This method would allow gene expression profiling of individual cells to better understand population heterogeneity and to distinguish between distinct cell subpopulations to increase the purity of desired cell lines (Poulin et al., 2014; La Manno et al., 2016; Reid and Wernisch, 2016; Lang et al., 2019; Tiklova et al., 2019). However, to achieve this, a specific set of cellular and gene regulatory network contexts have to be determined (as mentioned in the Assessment of the Efficacy of Cell Transplants With Immunostaining Characterization section). Although the presence of the PITX3 gene expression in adult mDA neurons suffices the criteria (Smidt et al., 1997), PITX3 was later shown to be present in both TH-positive and TH-negative cells (Tiklova et al., 2019). In the same study, single-cell RNA sequencing (scRNAseq) analyses were used to distinguish between several mDA subtypes with gene targeting. Moving forward, providing key proof-of-concept in utilizing scRNAseq as a tool for quality control would be the future for cell replacement therapies.

### Assessment of the Efficacy of Cell Transplants With Imaging

Last, concurrent with the high demand for the optimization of cell graft visualization in PD, growing emphasis has been placed on enhancing the sensitivity and precision of the spatiotemporal resolution of functional neuroimaging. En route to successful cell transplantation as a therapeutic regenerative method for Parkinson’s disease, neuroimaging techniques have to be employed for better patient care. Some key features required to elucidate the therapeutic efficacy of transplanted cells for clinical diagnostics are (1) innervation, (2) survival, (3) differentiation, and (4) functional biochemistry composition. Furthermore, it is crucial that these imaging techniques are time efficient, safe, non-invasive, and allow repeated measures in an individual to determine longitudinal post-operative progression in patients with cell transplantation (Barrow et al., 2015; Ramos-Gomez et al., 2015). In this section, we summarize the pros and cons of current imaging modalities used in tracking cell grafts in PD and their respective biomarkers (Table 2).

**TABLE 2:** Imaging modalities used in cell transplantation for PD.

**Modality**	**Purpose**	**Biomarkers**	**Measure**	**Advantages**	**Disadvantages**	**Pre-clinical**	**Clinical**
Magnetic resonance imaging (MRI)	Structural changes of brain tissue (i.e., cerebral atrophy)	Para-Gadolinium (III) (Gd^3+^)/Manganese (Mn^2+^) OR Superparamagnetic iron oxide (SPIO)	Gray matter volume OR Neuronal activity	• Repetitive measurements on the same individual • Full temporal profile of cell dynamics •↑ Tissue contrast • Microstructural analysis • Biodegradable labels (biocompatibility) • Required for PET data processing and analysis •↑ Availability in clinics	•↓Spatial resolution • Quantification of signal intensity changes with disease progression has to be optimized •↓Functional readout •↓Sensitivity • Cells have to be labeled prior to transplantation • Signal dropout • Limited normative database in clinics	Sykova and Jendelova, 2007; [Bibr B191][Bibr B162]; Wang et al., 2015; Malloy et al., 2017; Perez-Bouza et al., 2017	Piccini et al., 2005; Mendez et al., 2005; Morizane et al., 2013; Son et al., 2016
Single-photon emission computed tomography (SPECT)	Integrity of nigrostriatal dopaminergic pathways (presynaptic function of striatal neurons)	^123^I-*N*-ω-fluoropropyl-2β-carbomethoxy-3β-(4-idophenyl) nortropane (^123^I-FP-CIT/^123^I-ioflupane)/ 123I-IPT	Binds to striatal dopamine transporters (DAT)	•↑ Kinetics •↑ Selectivity Compatible with levodopa treatment •↑ Tissue penetration •↑ Half life Quantitative Readily available Repeated scanning	•↓ Specificity in diseases that causes loss of presynaptic dopamine neurons	N.A.	Pogarell et al., 2006; Politis et al., 2011; Son et al., 2016
Positron emission tomography (PET)	Functional readings of dopaminergic and non-dopaminergic systems in relation to pathogenesis and pathophysiology of PD	[^18^F]FDOPA/ [^18^F]Fallypride/ [^18^F]FBCTT/ [^11^C]-raclopride/ [^11^C]DASB/ [^11^C]PE2I/ [^11^C]CFT [^11^C]DTBZ [^11^C]PK1119/ [^11^C]-DAS	Aromatic amino acid decarboxylase (AADC—dopamine synthesis capacity and storage)/DA release (binds to striatal post-synaptic D2 receptors)/ 5-HT transporter (Pre-synaptic 5-HT terminal integrity and detection for serotoninergic neurons)	•↑ Sensitivity in differential detection of motor severity •↑ Tissue penetration •↑ Predictive value Correlates with motor progression over time	↓Half life ↓Precision (indirect measurement of dopamine synthesis) ↓Signal production •↓ Socioeconomic burden •↑ Radiation	Muramatsu et al., 2009; Emborg et al., 2013; Hallett et al., 2015; Goggi et al., 2020	Lindvall et al., 1990; Peschanski et al., 1994; Freeman et al., 1995; Wenning et al., 1997; Brundin et al., 2000; Piccini et al., 2000, 2005; Freed et al., 2001; Olanow et al., 2003; Mendez et al., 2005; Ma et al., 2010; Morizane et al., 2013

*Relative representation ↑, high; ↓, low.*

Magnetic resonance imaging (MRI) is a popular method for examining brain tissue morphology that uses strong magnetic fields coupled with contrast agents such as paramagnetic contrast agent (Gadolinium [III] [Gd^3+^], Manganese [Mn^2+^]), perfluorocarbons, or superparamagnetic iron oxide (SPIO) despite its challenges in differentiating tissues with structures that naturally emits low MRI signals like bones. Its biggest advantage is its superior spatial resolution, non-invasiveness, and relatively cost efficiency compared to other neuroimaging methods discussed below. Various lines of evidence strongly suggest the reliability of MRI in visualizing prelabeled transplanted cells such as ESCs (Sykova and Jendelova, 2007), fetal rat cortical cells (Hawrylak et al., 1993), and fetal striatal tissues (Norman et al., 1992) in rats. Furthermore, MRI has been used to evaluate edema and inflammation in tissues surrounding cell-transplanted sites in mice and primates (Anderson et al., 2005; Iwanami et al., 2005). It is important to note that false MRI signals may result from the residual build-up of SPIO nanoparticles released from dead transplanted cells and engulfed by macrophages and activated microglia (Amsalem et al., 2007; Liu and Frank, 2009; Cupaioli et al., 2014; Ramos-Gomez and Martinez-Serrano, 2016). Additionally, cells prelabeled with contrast agents prior to transplantation may show diluted and faded contrast over time as cells proliferate within the transplanted site, which may lead to a reduction in signal. Finally, MRI technology is predominately used in multimodality neuroimaging of cell transplantation by combining both structural and functional readouts for the improved refinement of clinical diagnostics. To this end, it could be coupled with the high sensitivity but low-resolution bioluminescence imaging (Tennstaedt et al., 2013), an economical and non-invasive technique using enzymatic chemiluminescence that allows full temporal live tracking of viable transplanted grafts.

Single-photon emission computed tomography (SPECT) is a type of nuclear imaging technique that utilizes specific gamma-emitting isotopes (compounds derived from cocaine that bind to the dopamine transporter) to analyze the integrity of the nigrostriatal DA pathway in PD (Son et al., 2016). SPECT biomarkers allow for the detection of presynaptic neuronal degeneration (Marshall and Grosset, 2003) and D2-type post-synaptic receptor density (Thobois et al., 2001). The clinical utility of such metabolic and neurochemical changes in PD is reviewed by Wang et al. (2012). To improve the diagnostic accuracy of SPECT imaging, current studies have employed the combined evaluation of both pre- and post-synaptic measurements through striatal dopamine transporters (DAT) and dopamine D2 receptor analysis, respectively (Koch et al., 2007). Further refinements must be made for SPECT imaging modality to be able to differentiate diseases with impairments in presynaptic DA neuronal survival such as PD, progressive supranuclear palsy, multiple system atrophy, and others (Bajaj et al., 2013). Also, potential leakage of radiotracers into adjacent cells resulting in diluted signals during cell proliferation has to be rectified. More importantly, the optimal concentration of tracers must be determined to avoid tissue damage due to exposure to toxic radioactive reagents. One disadvantage of this technique is its inability to examine cell survival and function.

Positron emission tomography (PET) is also a common imaging tool that employs specific radionuclides to elucidate the functional consequences of transplantation on the DA system in the brain, such as receptor distribution, metabolic activity, and inflammation (Visnyei et al., 2006). The measurement of aromatic L-amino acid decarboxylase activity using [^18^F]FDOPA is regarded as the gold standard to examine DA function and disease severity in Parkinson’s disease (Morrish et al., 1996; Punal-Rioboo et al., 2009), also shown in PD non-human primate model (Muramatsu et al., 2009; Emborg et al., 2013; Hallett et al., 2015) and clinical reports (Lindvall et al., 1990; Peschanski et al., 1994; Piccini et al., 2000, 2005; Ma et al., 2010) (refer to citation in Table 2). PET images can also be used in conjunction with SPECT data to further evaluate the negative association between striatal DAT and motor severity (Shih et al., 2006; Wu et al., 2014). Interestingly, recent clinical studies have shown that [^11^C]PE2I has higher predictive value and sensitivity toward the differential detection of motor impairments than [^18^F]DOPA imaging; hence, [^11^C]PE2I could be a prospective biomarker to investigate novel interventions (Fazio et al., 2015; Li et al., 2018). PET would be advantageous for studying the early maturation of cells transplanted *in vivo* and for follow-up examinations months after cell transplantation. A comprehensive and concise review on the development of functional neuroimaging is discussed in the cited works (Zheng et al., 2017; Helmich et al., 2018).

With no doubt, one of the most understudied limitations in neuroimaging is in deciphering the complexity of neuropathological overlap and clinical heterogeneity in the progression of individual neurological diseases. Improvements in bioimaging tools, such as the identification of specialized biomarkers for specific cell types to evaluate differential functional signatures, are important to circumvent the high level of variation in the prognosis of PD and its management by patients. In addition, the paucity of imaging modalities available for quantitation and of their respective analytical tools continues to hinder the further development of cell-based therapeutics toward clinically competitive treatments for PD. As discussed above (also refer to Table 2), we cannot rely on a single imaging technique for clinical diagnosis especially post-transplantation; thus, researchers are actively searching for the development of multimodality imaging (Waerzeggers et al., 2008) along with the identification of novel biomarkers and tracers to escalate the accuracy of post-operative care. A better understanding of neuroanatomical and pathophysiological processes would be highly advantageous for cell-derived therapeutics.

## Clinical Trials for Stem Cell-Derived DA Neuron Transplantation in Parkinson’s Disease

**FIGURE 1:**
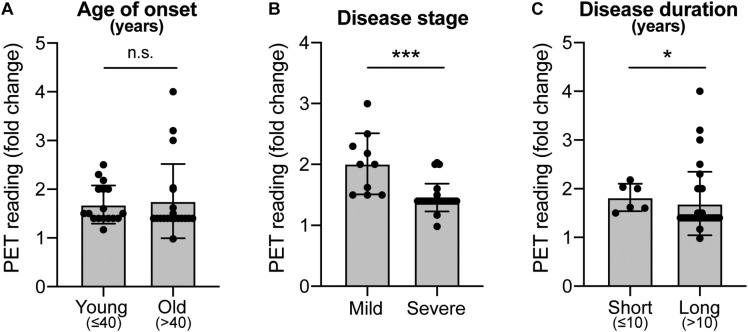
Systematic analysis of various factors associated with clinical outcomes using positron emission tomography (PET) readings of Parkinson’s disease (PD) patients with fetal ventral mesencephalic (fVM) cell transplantation. Statistical comparison was performed on various parameters against fold change of PET readings pre- and post-transplantation. **(A)** Age on onset: old (> 40 years) vs. young (≤ 40 years) PD patients. **(B)** Disease stage in mild and severe conditions. **(C)** Disease duration: long (> 10 years) vs. short (≤ 10 years). Student *t*-test, *p < 0.05, ***p < 0.001.

**FIGURE 2:**
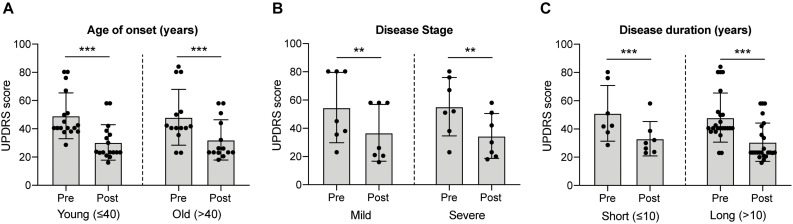
Systematic analysis of various factors associated with clinical outcome in fVM cell transplantation in PD patients using UPDRS motor scores. Statistical comparison was performed on varying parameters against the Unified Parkinson Disease Rating Scale (UPDRS) motor scores of pre- and post-transplantation. **(A)** Age on onset: old (> 40 years) vs. young (≤ 40 years) PD patients. **(B)** Disease stage in severe vs. mild condition. **(C)** Disease duration: long (> 10 years) vs. short (≤ 10 years). Two-way ANOVA, Sidak’s multiple comparisons test, **p < 0.005, ***p < 0.001.

Historically, fVM cell transplantation showed varied outcomes in human clinical trials (Table 3) (Freed et al., 2001; Olanow et al., 2001, Olanow et al., 2003, Redmond et al., 2001; Barker et al., 2013). A double-blind study of bilateral injection of fVM transplantation and sham surgery into the putamen was first performed in 19 PD patients by Freed and colleagues in 2001 (Freed et al., 2001). Interestingly, only younger age groups showed clinical improvements compared to the sham control (Freed et al., 2001). Using available data extracted from individual clinical papers cited in Table 3, we have performed systematic statistical analysis of the clinical outcomes of PD patients with fVM transplantation against various parameters, namely, age of onset (old, > 40 years vs. young, ≤ 40 years), disease stage (severe vs. mild), and disease duration (long, > 10 years vs. short, ≤ 10 years). The fold change of PET readings post-transplantation from the baseline reading of individual patients was used to access graft survival. We show that graft survival is independent of the age of disease onset (Figure 1A) but is dependent on variations in disease stage (Figure 1B) and the length of disease duration (Figure 1C), where better graft survival was observed in mild stage PD and patients with shorter disease duration (≤ 10 years). Moreover, we used the Unified Parkinson Disease Rating Scale (UPDRS) motor scores to examine clinical improvements post-transplantation of PD patients in various factors (Figure 2). We have demonstrated that in all three parameters (as mentioned above), PD patients with fVM transplantation have shown significant clinical improvements (correlated to the decrease in UPDRS motor scores) post-transplantation. Also, comparison between post-transplantation within each parameter (i.e., old vs. young or severe vs. mild or long vs. short) showed no significant differences. In summary, although clinical improvements can be observed throughout the wide spectrum of PD patients with fVM transplantation (Figure 2), the optimal condition with the most potential could be seen in mild stage PD patients with short disease duration (Figure 1).

**TABLE 3:** Summary of clinical studies in cell transplantation for PD.

**Year of Publication**	**Patient info**				**Results**			**No. of patients**	**Lewy body in grafted cells**	**References**
	**Age**	**Disease stage**	**Disease duration (years)**	**Follow-up (years)**	**Cell type**	**Graft survival**	**Clinical improvement**			
1989	48–55	Severe	14	0.5	fVM	N.A.	No	2	N.A.	Lindvall et al., 1989
1990	49	Severe	13	0.5	fVM	Yes	Yes	1	N.A.	Lindvall et al., 1989
1992	30–43	Severe	6	2	fVM	Yes	Yes	2	N.A.	Widner et al., 1992
1992	N.A.	Severe	N.A.	1.5	fVM	Yes	Yes	4	N.A.	Spencer et al., 1992
1992	50–60	Mild	8–11	1	fVM	Yes	No	2	N.A.	Sawle et al., 1992
1994	N.A.	N.A.	N.A.	3	fVM	Yes	Yes	2	N.A.	Lindvall et al., 1994
1994	N.A.	Severe	N.A.	1,1.5	fVM	Yes	Yes	2	N.A.	Peschanski et al., 1994
1995	39–61	Severe	8–22	0.5	fVM	Yes	Yes	4	N.A.	Freeman et al., 1995
1995	59	Severe	8	1.5	fVM	Yes	Yes	1	N.A.	Kordower et al., 2008
1997	43–58	N.A.	5–12	1–6	fVM	Yes	4 Patients effective	6	N.A.	Wenning et al., 1997
1999	69	Severe	9	10	fVM	Yes	Yes	1	N.A.	Piccini et al., 1999
2000	41–68	Mild-Severe	11–15	1.5–2	fVM	Yes	Yes	5	N.A.	Brundin et al., 2000
2001	34–75	Severe	14	1	fVM	Yes	Effective in younger patients	19	No	Freed et al., 1990
2002	52.0 ± 7.0	Mild-Severe	11.9 ± 2.2	11	fVM	N.A.	Not clear	14	N.A.	Hagell et al., 2002
2003	30–75	Severe	N.A.	2	fVM	Yes	Effective in milder patients	23	N.A.	Olanow et al., 2003
2005	54.1 ± 9.2	Mild	13 ± 2	2	fVM	N.A.	Not clear	9	N.A.	Piccini et al., 1999
2005	59,69	N.A.	11,15	3–4	fVM	Yes	Yes	2	No	Mendez et al., 2008
2008	N.A.	N.A.	N.A.	9–14	fVM	Yes	N.A.	5	No	Mendez et al., 2008
2008	61	Severe	22	14	fVM	Yes	Effective in initial 10 years	1	Yes	Kordower et al., 1995
2009	57	Mild	11	5	NPC	Yes	Effective in initial 3 years	1	N.A.	Levesque et al., 2009
2010	65	Severe	N.A.	12,16	fVM	Yes	N.A	1	Yes	Li et al., 2010
2011	69, 65	Severe	14, 12	22,12	fVM	Yes	No, Yes	2	Yes	Kurowska et al., 2011
2014	49,54	N.A.	10,12	18,15	fVM	N.A.	Yes	2	N.A.	Kefalopoulou et al., 2014
2016	59	N.A.	9	24	fVM	Yes	Effective in initial 14 years	1	Yes	Li et al., 2018
2017	55	Severe	8	16	fVM	Yes	No	1	Yes	Kordower et al., 2017
2020	69	Severe	10	2	iPSC	Yes	Yes	1	N.A.	Schweitzer et al., 2020

In line with our data, the high prevalence of long-term graft survival with low to no immune response in the majority of fVM recipients could be represented for future/ongoing stem cell-based clinical trials as a basis for host tissue innervation and reconnection to host DA circuitry. It is to note that occasional appearance of graft-induced dyskinesia cannot be attributed to cell transplantation as of date, as there are very limited follow-up studies. Upcoming clinical studies must include detailed surgical procedures, characterization of PD hallmarks such as α-synuclein-positive Lewy bodies, ubiquitin expression, and imaging analysis for F-DOPA uptake in graft region in addition to clinical observations. It is believed that the differences in quality and heterogeneity in the transplanted cells, patient selection, and surgical methodologies could have been the reason for failures in some trials. The current status of the TRANSEURO trial (NCT01898390), a large collaboration between the European Union multicenters of fetal nigral cell transplantation, which started in 2012, has grafted 11 young patients with early-stage PD in Cambridge, 2019, and will be subjected to clinical observations for 36 months post-surgery, which is estimated to be completed in early 2021.

With the improvement in the human DA neuron differentiation protocol (Nolbrant et al., 2017), more authentic midbrain DA neurons can now be derived from ESCs or iPSCs *in vitro*. These more defined ESC/iPSC-derived DA neurons show satisfactory therapeutic effectiveness in PD animal models (Studer, 2017), which has led to new waves of initiatives for cell transplantation in PD patients. Furthermore, with ES-derived DA neuronal transplantation being equipotent (Grealish et al., 2014) to that of the current gold standard for PD cell therapy (Li et al., 2016), stem cells rather than fetal neurons hold high expectation in the near future. However, we must bear in mind that animal models cannot fully reproduce human PD. Confounders, including aging, disease duration, disease severity, diabetes, and depression, should be taken into account when cell therapy is translated from preclinical models to clinical trials (Aarsland et al., 2011; Athauda et al., 2017; Henchcliffe and Parmar, 2018). Currently, ongoing clinical trials of the GForce-PD Consortium include European-based STEM-PD trial, NYSTEM trial, CiRA trial;^2^ Cyto Therapeutics Pty Limited founded trial (NCT02452723), and the Chinese Academy of Sciences founded trial (NCT03119636) lead by Qi Zhou. STEM-PD trial was designed to use GMP-grade hESCs as the clinical cell source, employing full GMP-grade production procedure (Kirkeby et al., 2017b), and transplanting 100,000 TH^+^ D16 mDA progenitors per graft as a target dose. In contrast, CiRA was designed to develop clinical-grade DA cell therapy from autologous iPSCs taken from PD patients (Barker et al., 2017). More recently, iPSC-derived dopamine progenitor cells have been bilaterally injected into a 69 year old PD patient and have demonstrated signs of improvements in motor assessment 24 months post-surgery (Schweitzer et al., 2020). It is interesting to note that clinical improvements were significantly associated with the right (second) surgical procedure than the left. One explanation would be the improved procedural efficiency including shorter time taken from cell harvest to implantation. Further double-blind studies will be essential to better understand the full potential of iPSC-derived dopamine cells in PD.

Notably, neural progenitor stem cells (NPCs) are an alternative cell source for cell replacement strategy. These multipotent cells can self-renew and differentiate into all mature neural cells in the CNS in large quantities (Ribeiro et al., 2013). The first autologous differentiated neural stem cell clinical trial was conducted using tissue samples collected in the prefrontal cortical and subcortical region along the trajectory of the electrode implant prior to further expansion and differentiation. The patient showed clinical improvements during the first 3 years post-transplantation with subsequent decline back to baseline by the fifth year (Levesque et al., 2009). Ongoing clinical trials sponsored by NeuroGenerations involves 12–20 PD patients at the age of 35–85 years (Hoehn and Yahr stage III or IV) with an estimated completion date by 2021 (NCT03309514, NCT01329926).

Another factor we need to consider for PD cell transplantation clinical trials is the patient stratification. Since aging-induced BBB leakage can lead to infection and inflammation, age can greatly compromise the survival of DA neurons. Moreover, cell transplantation is usually less effective in patients with a longer and more severe disease progression (Barker et al., 2017). Discrepancies were also found in some patients with substantial survival of grafted DA neurons but no beneficial behavioral improvements (Barker et al., 2013, 2015a, 2017; Barker, 2014). These observations possibly indicate the degeneration of other brain systems, especially the post-synaptic component of the DA system. Thus, DA neuron transplantation clinical trials initiated by various organizations only include patients who 1) are younger than 65 years old, 2) have a disease duration of less than 10 years, and 3) are in the early stage of the disease (Kirkeby et al., 2017b; Studer, 2017; Takahashi, 2017). Moving forward, the identification of these confounders would be helpful for clinicians to be able to better stratify PD patients and suggest the most suitable treatment strategy for each patient.

## Conclusion and Future Directions

We have highlighted four different types of cell sources and have addressed their pros and cons to better understand the characteristics of individual cell types and have also provided detailed analysis of the discrepancies observed in clinical outcomes of PD patients. This also includes methodologies in cell type specification and various imaging modalities. The emphasis on cell line availability, quality, and ability to innervate into host tissues, develop into functional A9 DA neurons, which would efficiently repair the host DA system is of topmost importance. Furthermore, long-term survivability for years after surgery without graft-induced dyskinesia or immune rejection by the host are some safety requirements and is the key to successful translation into large-scale therapeutic application and also in biomedical research to better recapitulate *in vitro* disease models. To avoid further inconsistencies in clinical results, we need to ensure standardization across several aspects of transplantations, including tissue preparation for engrafts, surgical technique, patient selection, immune therapy, synaptic integration capacity of cells transplanted into the human brain, and ways to bypass ethical issues revolving around cell transplantation in humans and the usage of ESCs (more details can be found in Barker et al., 2015b). The need to develop minimal, but precise, surgical techniques and achieving a better understanding of striatal functions are necessary, and these improvements could be accomplished by acquiring higher-resolution diagnostic imaging with heightened specificity for targeted graft placement and post-op observational study. Clinical endpoint observations from aforementioned ongoing clinical trials would pave a way to develop a coherent and systematic blueprint for therapeutic strategies such as improved surgical methodologies, optimized and standardized protocols, development of appropriate safeguards, and objective long-term outcome measures. The field of stem cell-based therapies for PD has entered into an exciting era, and we believe there is greater optimism that neurotransplantation may provide a viable option for treatment of PD in the future.

## Author Contributions

SEJ, LQ, and LZ have drafted the manuscript with inputs from LLC and E-KT. All authors contributed to the article and approved the submitted version.

## Conflict of Interest

The authors declare that the research was conducted in the absence of any commercial or financial relationships that could be construed as a potential conflict of interest.

## Footnotes

1 https://www.mskcc.org

2 https://upload.umin.ac.jp/cgi-open-bin/ctr_e/ctr_view.cgi?recptno=R000038278
